# Influence of Different Hot Runner-Systems in the Injection Molding Process on the Structural and Mechanical Properties of Regenerated Cellulose Fiber Reinforced Polypropylene

**DOI:** 10.3390/polym15081924

**Published:** 2023-04-18

**Authors:** Jan-Christoph Zarges, André Schlink, Fabian Lins, Jörg Essinger, Stefan Sommer, Hans-Peter Heim

**Affiliations:** 1 Institute of Material Engineering, Polymer Engineering, University of Kassel, 34125 Kassel, Germany; 2 Günther Heisskanaltechnik GmbH, 35066 Frankenberg (Eder), Germany

**Keywords:** viscose fiber, natural fiber composites, hot runner system, injection molding, fiber length, fiber orientation, X-ray microtomography analysis, mechanical properties, dynamic image analysis

## Abstract

The increasing demand for renewable raw materials and lightweight composites leads to an increasing request for natural fiber composites (NFC) in series production. In order to be able to use NFC competitively, they must also be processable with hot runner systems in injection molding series production. For this reason, the influences of two hot runner systems on the structural and mechanical properties of Polypropylene with 20 wt.% regenerated cellulose fibers (RCF) were investigated. Therefore, the material was processed into test specimens using two different hot runner systems (open and valve gate) and six different process settings. The tensile tests carried out showed very good strength for both hot runner systems, which were max. 20% below the reference specimen processed with a cold runner and, however, significantly influenced by the different parameter settings. Fiber length measurements with the dynamic image analysis showed approx. 20% lower median values of GF and 5% lower of RCF through the processing with both hot runner systems compared to the reference, although the influence of the parameter settings was small. The X-ray microtomography performed on the open hot runner samples showed the influences of the parameter settings on the fiber orientation. In summary, it was shown that RCF composites can be processed with different hot runner systems in a wide process window. Nevertheless, the specimens of the setting with the lowest applied thermal load showed the best mechanical properties for both hot runner systems. It was furthermore shown that the resulting mechanical properties of the composites are not only due to one structural property (fiber length, orientation, or thermally induced changes in fiber properties) but are based on a combination of several material- and process-related properties.

## 1. Introduction

Natural fiber composites (NFC) with polypropylene (PP) as matrix material and short natural fibers for reinforcement are increasingly used in series production, e.g., in the field of automotive parts and other engineering applications [1,2,3]. The reason for this is the increasing demand for renewable raw materials and the possibility to combine good mechanical properties with the lightweight potential of low-density fibers [4,5]. A large number of studies regarding the processing and characterization of NFC used compression molding for specimen production [6,7,8,9], while injection molding tends to be more important for industrial applications, especially for the production of serial parts. In most natural fiber composites, polyolefins are used as matrix material, and natural plant fibers are used for reinforcement [7,8,10,11]. The reason for using polyolefins is their comparatively low processing temperature of approx. 200 °C, which allows processing with almost no thermal degradation of the sensitive natural fibers (NF). Degradation, the rate of which depends strongly on the exposure temperature, starts at approx. 170 °C, depending on the cellulose content of the NF [12,13], and results in a reduction in mechanical properties [14,15,16,17,18]. The pure cellulose fibers are thermally more stable and show significant material degradation in thermogravimetric analyses only from 240 °C due to a measured loss of mass [19,20,21,22].

A major disadvantage of NFC is the deviating geometrical and mechanical properties of the natural fibers used, which makes it considerably difficult to predict the fracture and failure behavior of NFC [2,23]. For this reason, regenerated cellulose fibers (RCF) have been used as reinforcement in addition to plant fibers (hemp, flax, jute, sisal, etc.) in previous studies. RCFs, also known as viscose or rayon, produced by the viscose process have very constant geometric and mechanical properties in addition to their lower density compared to conventional glass fibers and the significant lightweight potential associated with them [24,25,26]. Another reason for using RCFs as reinforcement is their higher elongation at break (approx. 13%) compared to that of glass fibers (approx. 2%), resulting in a more ductile, less spontaneous, and more predictable failure behavior of the composites [9,27]. This toughness and the good mechanical properties of RCF composites in general can be attributed to the fiber length distribution and fiber-matrix adhesion [28,29,30]. Due to the lower bending stiffness of RCF compared to GF, the RCF is less shortened by the shear stresses in the compounding and injection molding process and thus still exceed the critical fiber length in some cases even in the test specimens or components, while the lengths of GF in the components are usually significantly below the critical fiber length. This leads to a good reinforcement effect of the RCF and at the same time to a good toughness due to friction-intensive fiber pullouts at failure [9,30,31,32].

The above-mentioned properties of RCF lead to a significant increase in mechanical properties, especially notched impact strength, and fracture toughness [12,33,34]. More specifically, compared with glass fiber reinforced composites (GFC), the values of notched impact strength are about four times higher at the same fiber weight content, and the values of fracture toughness are about three times higher [31,35], while the density is 20% lower.

With regard to the structure, in particular, the fiber orientation, which is induced by the injection molding process, the RCF composites, similar to all other short glass fiber reinforced thermoplastics, are significantly influenced by the injection molding parameters. As a result, the short fiber reinforced thermoplastics exhibited locally different microstructures, such as crystallinity [36] and fiber orientations [37,38,39], due to the processing influences, which can have a significant influence on mechanical and fatigue properties [36,39,40,41,42,43,44]. Several publications have characterized the influence of fiber orientation in injection molded composites, showing that the more fibers oriented in the loading direction, the better the mechanical properties [44,45,46,47]. With regard to the process influences during injection molding, a significant influence of the melt and mold temperature as well as the volume flow rate could be shown. It was demonstrated that higher melt temperatures and volume flow rates have a positive effect on the strength, stiffness, and fatigue properties of the resulting GFC due to the higher number of fibers oriented in the direction of loading [47,48].

In order to make the natural or viscose fiber-reinforced plastics competitive as well as to increase efficiency and reduce material waste, as mentioned above, it must be possible to process the RCF composites in the injection molding process even with hot runner systems. This, however, is complicated by the thermal sensitivity of RCF and the longer dwell time of the melt in the hot runner at a higher temperature, which negatively affects the mechanical properties of RCF, such as tensile strength and elongation at break [10,14,18,19,27,34]. At the same time, the small cross-sections in a hot runner system can lead to higher shear and thus to a significant reduction in RCF length [8,17,28,31]. Both result in a reduction of the mechanical properties of the RCF composites. In addition, the high fiber length combined with the flexural properties of the RCF could lead to a plugging of the narrow cross sections in a hot runner system, which would make a trouble-free series production considerably more difficult. 

As explained in detail here, RCF composites have not yet been processed with hot runner systems in series-production injection molding processes in previous investigations. The reason for this is that it was not clear until now to what extent processing is possible without exposing the RCF composites to excessive thermal and mechanical stress. The objective of this paper is to demonstrate the feasibility of this processing and to present a process-structure-property correlation. For this purpose, two different hot runner systems and a conventional cold runner system (as reference) are used, combined with different injection molding process parameters. In addition to mechanical properties, structural properties such as fiber lengths and orientations are characterized and presented in correlation to the process parameters. To illustrate the material differences of an RCF composite, reference composites of a glass fiber-reinforced PP are characterized in parallel.

## 2. Materials, Processing, and Characterization

### 2.1. Used Materials

The polypropylene PP 575P used as the matrix material, was provided by the company Sabic (Riyadh, Saudi Arabia). The PP used has a melt flow rate (MFR) of 10.5 g/10 min at 230 °C and 2.16 kg and a density of 0.905 g/cm³. According to the manufacturer’s datasheet, the processing temperature of the material is 200–225 °C, while the molecular weight distribution is given as broad. 

Chopped, regenerated cellulose fibers provided by the company Cordenka GmbH & Co. KG (Obernburg, Germany) with an average filament diameter of approx. 12 µm and an average initial length of approx. 2.3 mm was used for reinforcement. The fibers are produced by the viscose process, which is still considered the main large-scale RCF production process, although both the lyocell and carbamate processes are far less environmentally harmful than the viscose process because of the chemicals used [49,50]. Compared to the Lyocell fibers (Tencel), the rayon fibers from Cordenka have a significantly higher Young’s modulus and tensile strength (see Table 1). The pure cellulose fibers have only very short fiber lengths, which, compared to the Lyocell and rayon fibers, achieve only a very small strengthening effect in the composite [51,52,53].

The very good mechanical properties of RCF by Cordenka are also reflected in the mechanical properties of the composites, which were already shown in a large number of publications and independent of the matrix material. In addition to the good quasi-static properties of the RCF composites, this always led to a significant increase in the notched impact strength, both in comparison with glass fibers and other natural fibers [34,35,54,55,56,57]. The used RCF were coated with a PPL-sizing (aqueous polyvinyl alcohol solution) of approx. 10 wt.% by the manufacturer, which was applied to increase the pourability for use in a gravimetric feeding systems. The RCF exhibits a density of 1.5 g/cm³ and an elongation at a break of 13%, a Young’s modulus of approx. 22 GPa, and a tensile strength of approx. 800 MPa [58,59,60]. 

Regenerated cellulose fibers (RCF) are referred to as cellulose type II due to their molecular structure caused by the manufacturing process, which usually has larger non-crystalline regions [19,50,61,62]. A major difference from cellulose type I is its behavior in absorbing and releasing moisture. The tensile strength of cellulose type II (RCF fibers) increases with the release of water, e.g., residual moisture at higher temperatures, while the elongation at break decreases [62,63,64]. For that reason, the RCF was dried prior to compounding.

In addition to that, glass fibers CS 7952 provided by the company Lanxess AG (Cologne, Germany) with a diameter of 14 µm, an initial length of 4.5 mm, a density of 2.6 g/cm³ and a sizing suitable for polypropylene were investigated for reference purposes. 

### 2.2. Compounding

To compare the properties of the glass and cellulose fiber reinforced composites, compounds with 20 wt.% of each fiber were compounded using the twin screw extruder ZSE 18 HPE (Leistritz Extrusionstechnik GmbH, Nuremberg, Germany) that has a screw diameter of 18 mm and a process length of 40 D. The fiber content of 20 wt.% was chosen because 20 or 30 wt.% glass fibers are currently mainly used for reinforcement in technical components. Due to the significantly lower density of RCF (1.5 g/cm^3^) compared to glass fibers (2.6 g/cm^3^), this results in a significantly higher fiber volume content of RCF for the same fiber weight content. Since it was not clear at the beginning of the test series whether the high fiber volume content at 30 wt.% RCF could lead to a problem during processing with the hot runner systems, 20 wt.% RCF was used.

Prior to the compounding process, the cellulosic fibers were dried in an air convection oven until their moisture content was less than 1%. All materials were fed into the extruder via a gravimetric feeding system (Brabender Technologie, Duisburg, Germany). After compounding, the strand was cooled down on a discharge conveyer using compressed air before being pelletized to a length of approx. 3 mm by a Scheer SGS 25-E strand pelletizer (Maag Germany GmbH, Grossostheim, Germany).

The screw configuration was optimized regarding less shear stress in previous investigations. For the named reason the configuration only consists of conveying elements after the fiber feeding zone, which reduces the shear stress but still realizes a homogeneous distribution of the fibers. The screw speed was set to 200 rpm while the processing temperatures were also set to a lower and more gentle level (below 200 °C, see Table 2) to reduce the thermal load on the cellulosic fibers [31,35,51].

### 2.3. Injection Molding

Prior to the injection molding process, the compounds containing viscose fibers were dried using an air dryer TORO-systems TR–Dry–Jet EASY 15 (Gfk Thomas Jakob und Robert Krämer GbR, Igensdorf, Germany) for 4 h at 80 °C. 

**Figure 1:**
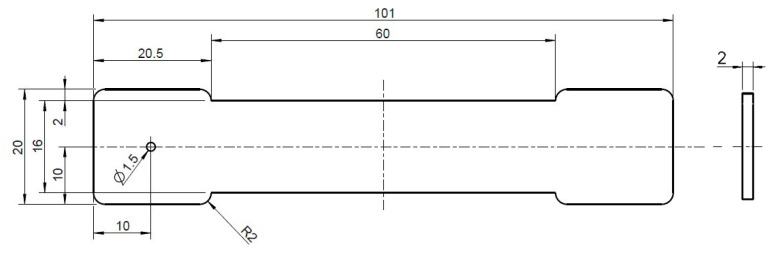
Schematic representation of the produced specimen with point gate of the hot runner systems.

The test specimens (see Figure 1) were produced on two different injection molding machines. In connection with the valve gate hot runner system, a hybrid injection molding machine from Engel VC200/80 Electric (ENGEL AUSTRIA GmbH, Schwertberg, Austria) was used. This machine is equipped with an electric injection unit and a hydraulic clamping unit. The clamping force is 500 kN, the screw diameter is 30 mm with a resulting max. metering volume of 85 cm³ and the used open machine nozzle has a diameter of 6 mm.

For comparison, test specimens were also produced with an open hot runner system, for which a hydraulic injection molding machine Arburg A270S (Arburg GmbH + Co KG, Loßburg, Germany) was used. This machine has a clamping force of 250 kN, and a screw diameter of 22 mm with a resulting max. metering volume of 30 cm³ and a machine nozzle with a diameter of 6 mm. The mold temperature on both machines was set to 40 °C and the temperatures of the plasticizing unit were set according to the following Table 3.

With both hot runner systems, the melt is injected via the point gate and spreads out from there in a circular shape in the cavity (see Figure 1).

After adjusting the process (Setting 01), the composites with cellulose fibers (PP 20RCF) were then subjected to successive increases in temperature and dwell time (see Table 4) in order to show the influence of the higher thermal load, which results from a combination of the temperature and the dwell time, on the structural and mechanical properties.

**Figure 2:**
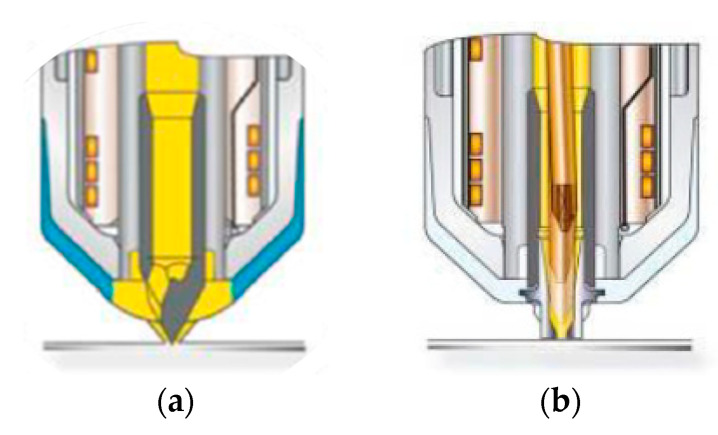
Schematic representation of the open hot runner system with tip (**a**) and the valve gate hot runner system (**b**).

In cooperation with Günther Heisskanaltechnik GmbH (Frankenberg, Germany) the following two hot runner systems (see Figure 2) were used to characterize the feasibility of processing cellulose fiber reinforced composites with different hot runner systems and their influence on the structural and mechanical properties of the cellulose and glass fiber reinforced plastics:

(a) Open hot runner system with tip (5SHF50) with a gate diameter of 1.5 mm
(b) Valve gate hot runner system (nozzle 6NHF50 LA-1.4; needle 3NHP175-1.4 (clamping force of the needle: 800 N)

Both nozzle typesthe fiber length distribution and orientation (see are designed with the Blueflow^®^ heaters by Günther Heisskanaltechnik. In conjunction with the two-part shaft (steel vs. titanium alloy with low thermal conductivity) of the nozzles, this results in a very homogeneous temperature profile in the nozzle and reduces the heat transfer from the nozzle to the mold. The BlueFlow^®^ technology involves heating elements that are manufactured on the basis of thick-film technology. Here, the dielectric layers and the heating conductor are applied under clean room conditions using the screen printing process. The manufacturer declares the following advantages compared to similar systems:

(a) Precise and homogeneous power distribution over the entire length of the nozzle
(b) Avoidance of temperature peaks in the melt-carrying material tube
(c) High power concentration in the front nozzle area.
(d) Rapid thermal reaction, thereby lower energy consumption

Due to the rapid thermal reaction of these heating elements, appropriate control technology must also be used. Therefore, a DPT control device from Günther Heisskanaltechnik GmbH was used for the tests. Experience shows that the homogeneous temperature profile of the applied hot runner and control technology is particularly suitable for the processing of thermally and shear-sensitive bioplastics or compounds with natural fibers in order to avoid thermal damage to the melt or the natural fibers.

In order to be able to quantify the influence of processing with the hot runner systems on the mechanical properties in a comparative manner, reference test specimens were produced with a cold runner system on a hydraulic injection molding machine Allrounder 320C Golden Edition (Arburg GmbH + Co KG, Loßburg, Germany) with a screw diameter of 25 mm and a clamping force of 500 kN. The cycle time was approximately 43 s, including a cooling time of 20 s.

### 2.4. Characterization

All composites were characterized while in a dry state and in a standardized climate (23 °C, 50% relative humidity).

#### 2.4.1. Tensile Test

Tensile tests were carried out at a speed of 5 mm/min according to EN ISO 527 using a UPM 1446 testing machine (Zwick Roell, Ulm, Germany) with a 10 kN load cell. During the tests, Young’s modulus, tensile strength, and elongation at break were evaluated. Five specimens were tested for each material.

#### 2.4.2. Color Measurement

Any thermal loads to the cellulose fibers resulting from processing lead to a darkening of the composites due to the degradation that occurs.

To objectively quantify this discoloration and its influence by individual process parameters, color measurements were carried out on the test specimens using the Ultra Scan Pro spectrophotometer (Hunterlab, Reston, VA, USA). This system uses the L*a*b*-color-model for color measurement, in which the brightness of a color is indicated by the L-value. The higher the L-value, the brighter the color. More precisely, an L value of 100 means that the color is white, while an L-value of 0 means a black color.

#### 2.4.3. Fiber Length Measurement

The resulting fiber length in the tensile specimen was measured using the dynamic image analysis QicPic R06 (Sympatec GmbH, Clausthal-Zellerfeld, Germany) with a Mixcel liquid dispersion unit. The fibers of representative parts of the specimens were separated from the matrix using xylene with a temperature of 80 °C for a duration of min. six hours. Afterward, the fibers were dispersed in isopropanol and filled into the liquid dispersion unit of the measuring system, which provided a constant flow of the dispersed fibers. This isopropanol flow with the fibers passed a cuvette with a thickness of 2 mm and a window at which a high-speed camera captured images of each fiber. Subsequently, the software Windox calculated the length and diameter of the fibers. The objective M7 with a resolution of 4.2 µm was employed, which realizes a minimal fiber size of 12.6 µm and a maximum fiber size of 8.66 mm. The distribution of the calculated fiber lengths is number-based (q0), which is well-suited for representing broad ranges of size distributions.

#### 2.4.4. X-ray Microtomography Analysis of the Composite Structure

For a structural characterization by X-ray microtomography (µCT), RCF-reinforced specimens produced with Settings 01, 02, 03, 05, 06 and the open hot runner system were selected. The intention of these analyses is to determine the correlation between fiber orientations within the parts and their mechanical properties.

High-resolution results have been obtained using an X-ray microtomograph Zeiss Xradia Versa 520 (Carl Zeiss, Oberkochen, Germany). These allow individual fibers to be examined separately and evaluated quantitatively. The measurements were performed at a voltage of 72 kV and a current of 83 μA using the 0.4x objective and no filter. The number of images acquired with an exposure time of 3.5 s for each image was 1601. Binning setting 1 resulted in a voxel size of 4.13 μm. These settings were chosen to obtain an image section in the center of the specimens of half the cross-section with an adequate voxel size. The subsequent reconstruction was performed with the Zeiss XMReconstructor software. A 3D data visualization and analysis software system Avizo 9.4 (Thermo Fisher Scientific, Waltham, MA, USA) with the XFiber extension for the quantitative analysis of fiber properties was utilized to generate the required data. After the preparation of the volume data, primarily the software modules “Cylinder Correlation” and “Trace Correlation Lines” were employed to detect the individual fibers regarding their fiber orientation with the settings from Table 5. The minimum continuation quality parameter was chosen in order to ensure that the resulting fiber lengths of the fiber tracing model have the same median as the fiber lengths of the QicPic fiber length measurements (see Section 2.4.3).

**Figure 3:**
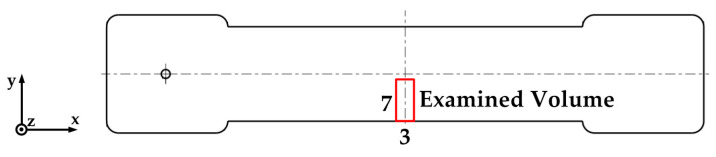
Position and size (in mm) of the examined sample volume by means of X-ray-micro-tomography (red).

The investigated volume within the samples is shown in Figure 3. Within this region, the orientation angle theta (Θ) was evaluated. It describes the angle between the *x*-axis and the yz-plane. In the case of Θ = 0°, a fiber is positioned exactly in the direction of flow respectively in the direction of the load during tensile tests, in the case of Θ = 90° perpendicular to this.

## 3. Results

### 3.1. Mechanical Properties

**Figure 4:**
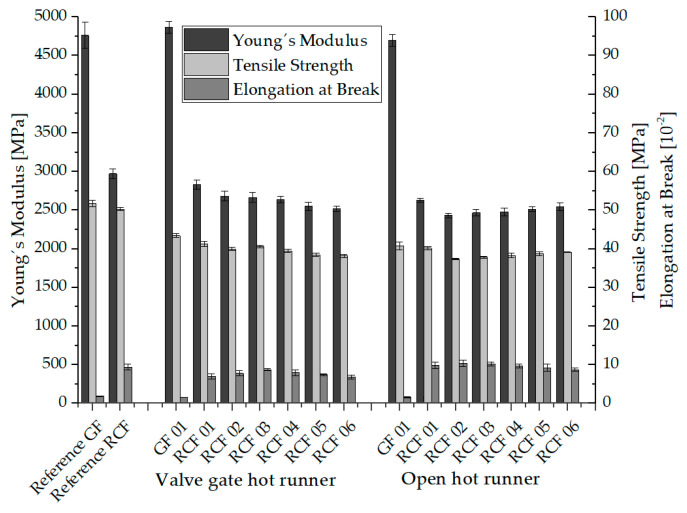
Mechanical properties of the RCF and GF reinforced composites.

The comparison of the mechanical properties of glass and cellulose fiber reinforced composites in Figure 4 initially shows the expected significantly higher stiffness and lower elongation at the break of the GF composites. 

Furthermore, with regard to the glass fiber reinforced composites, it can be seen that the values of stiffness (Young’s modulus) and elongation at break obtained during processing with the two hot runner systems are very close to those of the reference composites processed with a cold runner system. Regarding the tensile strength, a drop in the values of the GF composites of both hot runner systems of approx. 20% compared to the reference specimen is noticeable. The extent to which this drop is caused by damage to the structures, in particular a reduction in fiber length, by the hot runner systems is described with the characterized fiber length distributions in Section 3.3.

For the cellulose fiber reinforced composites, a reduction in Young’s modulus of 5% for the valve gate hot runner system and 10% for the open hot runner with tip is shown in comparison between the reference specimen and the specimen of RCF 01 (Setting 01). The tensile strength drops by 20% with both hot runner systems compared to the reference specimen. There are also further differences between the composites RCF 01 to RCF 06, which were processed with the valve gate hot runner system. For example, Young’s modulus and tensile strength decrease with increasing composite number. This can be explained by the combination of higher hot runner temperature and higher dwell time of the melt in the hot runner due to the higher cooling time in the injection molding process (see Table 4), which leads to higher thermal load and damage of the fibers. The decreasing elongation at break also indicates higher thermal damage to the fibers. The lowest tensile strength and stiffness values of specimen of RCF 06 are in total approx. 30% below the cellulose fiber-reinforced reference specimen.

The mechanical properties resulting from the different process settings can be attributed to the thermal properties of the RCF in addition to the fiber length distribution and orientation (see Section 3.3 and Section 3.4). Higher temperature exposure leads to degradation phenomena, which occur in RCF from about 220 °C due to the breaking of chemical bonds [19,65]. Overall, pure cellulose (e.g., RCF) exhibits higher heat resistance compared to conventional natural fibers [12,13,66]. With the onset of the degradation processes, the tensile strength of the RCF and especially the elongation at break decreases. In addition, the brittleness of the fibers and the associated higher number of fiber breaks in the process leads to a reduction in fiber lengths [67,68].

The tensile strengths and stiffnesses of the RCF-reinforced composites processed by means of an open hot runner system with tip are rather different. After a reduction in tensile strength (3 MPa) and stiffness (200 MPa) from batch RCF 01 to RCF 02, the values increase again up to batch RCF 06, although this cannot be described as a statistically significant change. At the same time, the elongation at the break of the composites decreases. In detail, this means that a higher melt temperature and the associated low viscosity in combination with a higher volume flow rate (at a shorter injection time) leads to higher tensile strengths and stiffnesses of the test specimens, since the fibers tend to be oriented in the flow direction and thus in the loading direction [41,47,69]. This will be validated with the results of the µCT analysis and the resulting fiber orientation in Section 3.4. The correlation between the shear rate and the viscosity of PP reinforced with glass and cellulose fibers has been shown in previous work. Here, a decreasing viscosity with a higher shear rate could be shown, whereby in particular the higher fiber volume fraction due to the RCF´s low density led to higher viscosities of the RCF composites overall [51,70].

**Figure 5:**
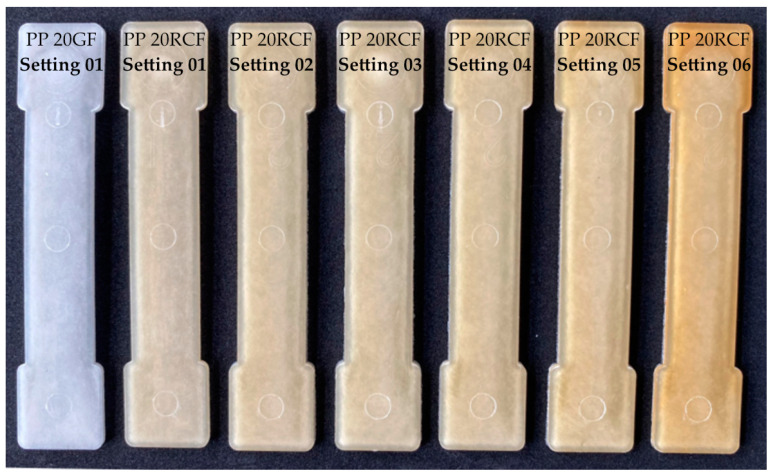
Representative specimens of PP 20GF (Setting 01) and PP 20RCF (Setting 01 to Setting 06).

The increasing dark coloration of the test specimens with increasing thermal loading (see Figure 5) is described by the results of the color measurement in Section 3.2, while the influence of the fiber length distribution and orientation is described in Section 3.3 and Section 3.4.

### 3.2. Discoloration

**Figure 6:**
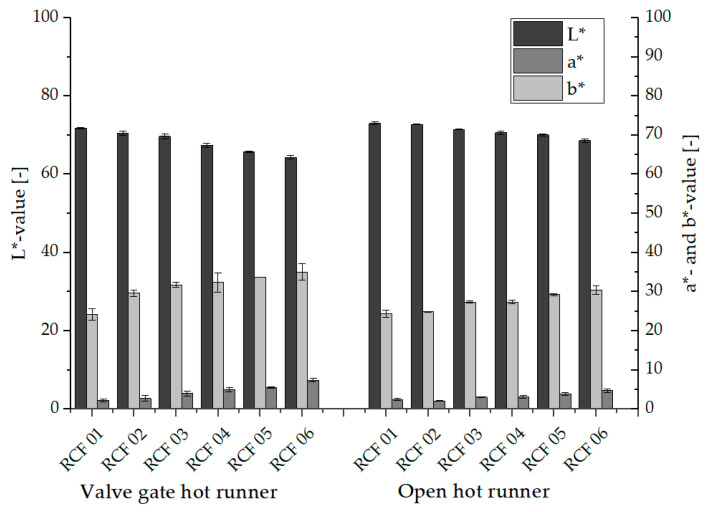
L*a*b*-values of the RCF und GF reinforced composites.

Figure 5 shows representative specimens processed with the settings of Table 4, while Figure 6 shows the results of the color measurement described in Section 2.4.2. 

It can be seen that in the tests with both hot runner systems, the composites show a lower L-value and thus a stronger dark coloration with the increasing number and thus with increasing thermal load. Cellulose is a polysaccharide and thus a sugar in which the process of caramelization takes place at higher temperatures (approx. 140 °C for saccharides). This process is accompanied by a darker coloration of the cellulose and thus also of the RCF, as well as the development of a roasted aroma (caramel odor). Since, depending on the thermal stability of the RCF, degradation processes do not yet occur immediately, the mechanical properties initially decrease only to a minor extent. With further increasing temperatures or longer dwell times, degradation of the RCF then leads to a reduction in tensile strength and elongation at break of the fibers. [18,27,35]. In addition to the decreasing L-value, an increase in the a-value and b-value can be seen with increasing thermal stress, which is indicative of an overall more intense coloration of the RCF composites.

### 3.3. Fiber Length Distribution

**Figure 7:**
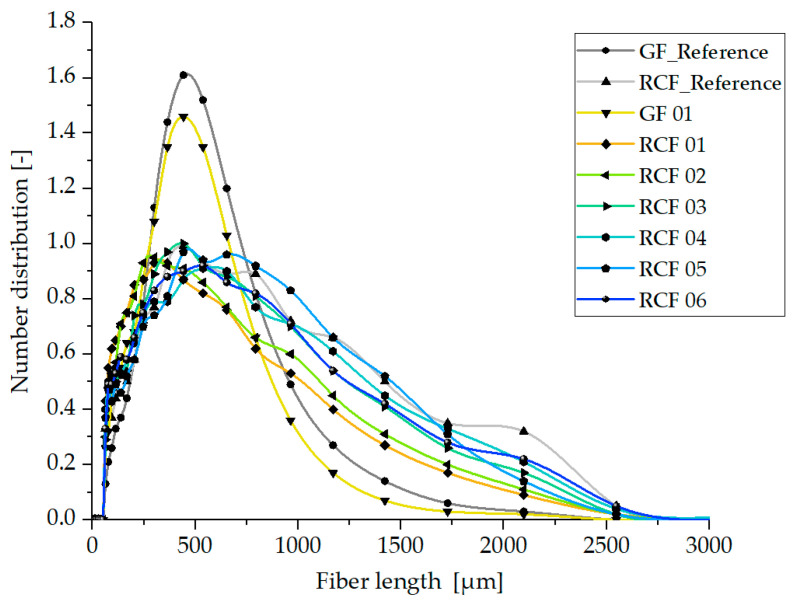
Fiber length distribution in specimen produced with the open hot runner system.

**Figure 8:**
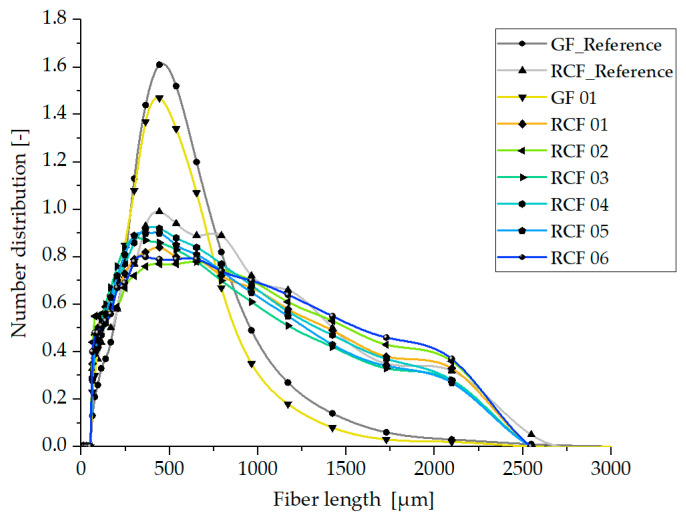
Fiber length distribution in specimen produced with the valve gate hot runner system.

The results of the fiber length distributions determined with the help of the dynamic image analysis system QicPic, described in Section 2.4.3, are presented and explained in the following. Figure 7 and Figure 8 show the fiber length distributions of the glass fiber reinforced PP and the results of the six parameter settings for the RCF reinforced PP processed with the hot runner system (see Table 4) as well as the two reference composites processed with the cold runner system.

The fiber lengths of the materials processed with the open hot runner system, shown in Figure 7, initially show the two distribution curves typical for GF and RCF. The glass fibers show a Gaussian distribution with a median of about 411 µm. A comparison of the two GF distributions shows that the fiber lengths of the material processed with the open hot runner are slightly below the values of the reference batch (median: 357 µm). Thus, there is shortening of the GF in the hot runner of approx. 15%, which is, however, sufficient to bring about the 20% reduction in tensile strength described in Section 3.1.

In contrast to the GF, the distribution of the RCF lengths does not turn out to be a Gaussian distribution. Due to the already described lower stiffness and higher elongation at break, the RCF are not shortened as much and are therefore present in greater numbers with higher lengths. This allows the good mechanical properties of these composites described above. Compared to the six hot runner composites, the reference composite has a similar fiber length distribution and only slightly more long fibers (10% higher median). Contrary to the assumption, this could be due to a similar shear in the cold runner geometry of the reference composite, which is different from that of the hot runner. The differences between the RCF reinforced composites of Settings 03 to 06 are very small and insignificant and can be attributed to minor differences in viscosity due to the process parameters from Table 4. In comparison, the RCF lengths of Settings 01 and 02 show lower values of fiber lengths, which can be attributed to the melt viscosity due to the low temperatures and dwell times and the resulting high viscosity (see Table 4).

Table 6 shows the values of the 10th, 50th and 90th percentile of the fiber length distribution of both hot runner systems for comparison.

Figure 8 shows the fiber length distributions of the materials processed with the valve gate hot runner system. The typical distribution curves for GF and RCF can also be clearly seen here. When comparing the two normal distributions of the glass fibers, the valve gate hot runner also shows only a slight shortening of the GF. Similar to the open hot runner system, there is a shortening of the GF of approx. 13%, which nevertheless leads to the 20% reduction in tensile strength described in Section 3.1.

The distribution of RCF lengths slightly deviates from that of the open hot runner. Contrary to the assumption the geometry and cross-sections of the valve gate hot runner does not result in a greater shortening of the RCF. Compared to the fiber length distribution of the open hot runner and the reference specimen processed with cold runner this is reflected in a larger number of longer fibers and a smaller number of shorter fibers. This result reflects very well the better mechanical properties (see Figure 4), in which the composites from the processing with valve gate hot runner show the better mechanical properties, which can now be attributed, at least in part, to the longer fiber lengths.

The comparison of the six process settings shows only very small differences in the fiber length distributions, so that the influence of the valve gate hot runner is greater than the influence of the process parameters used (see Table 4).

Based on the shown results, the reduction in tensile strength of RCF composite specimens from Setting 01 to 06 of both hot runner systems compared to the reference cannot only be attributed to the reduced fiber lengths but rather a combination with a reduction in tensile strength of the RCF due to thermal loading of the fibers. This will be discussed in more detail in the following sections.

### 3.4. Fiber Orientation

**Figure 9:**
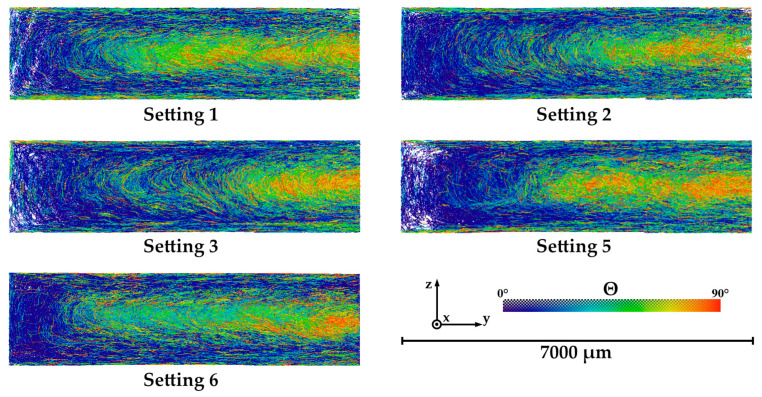
Illustrations of fiber orientations of RCF reinforced specimens produced with open hot runner system obtained by fiber tracing evaluation of the µCT data.

Figure 9 shows the fiber orientations of the specimens produced with different Settings and with open hot runner system, which were detected with the trace algorithm (see Section 2.4.4). It should be noted that the individual fibers are represented by lines in three-dimensional space and that white areas indicate that there are only few fibers. On the one hand, this can be explained by the lower fiber content in the edge areas of the sample. On the other hand, this area is located at the outer edge of the µCT measurement volume, where the image sharpness is lower, so that fewer fibers can be detected. Here, the fibers that are present in the flow direction (x-direction) and thus have an angle of Θ = 0° are colored blue. The fibers oriented perpendicular to the flow direction and thus aligned closer to the yz-plane (Θ = 90°) are shown in red. Each image represents the measuring area marked in Figure 3.

In this representation, the core of the sample is located on the right, a mold wall is positioned on the left, as well as at the upper and lower edges. The blue areas on the mold walls (edge regions) mark a high degree of orientation along the flow direction, while the fibers in the core region are rather unoriented or oriented perpendicular to the *x*-axis (red). The strong orientation of the fibers in the edge regions is due to classical layered models, which provide an explanation for the parallel orientation (0°) at high shear rates [71,72,73]. The fibers aligned in this way cool and freeze particularly quickly at the mold wall, so that this alignment is conserved. The core layers are influenced by the swell flow and lead to this (approximately) perpendicular alignment. The size of the respective areas can be attributed to the different process parameters within the settings used.

**Figure 10:**
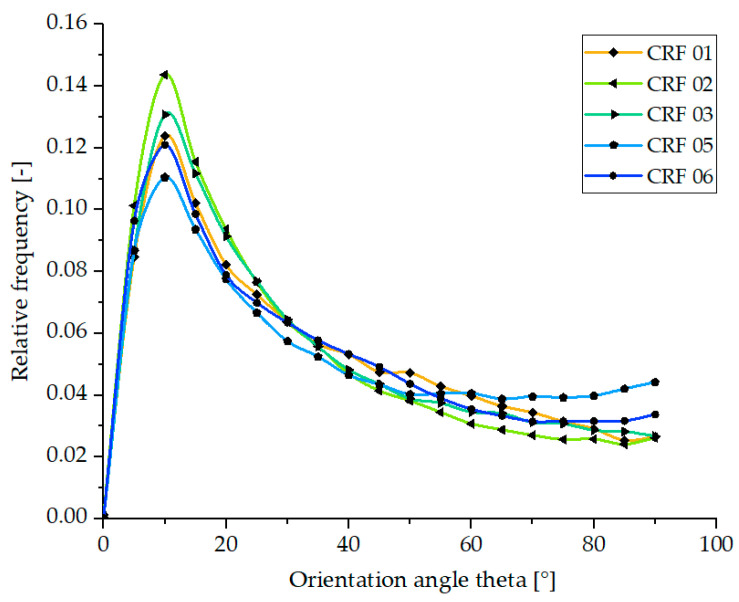
Quantitative plot of fiber orientations of RCF reinforced specimens produced with open hot runner system obtained by fiber tracing evaluation of the µCT data.

The frequency distributions of the angle Θ derived from fiber tracing are shown in Figure 10. Here, the test specimens of Settings 01, 02, and 03 show the highest values in the range of Θ = 5–15° and thus have the most fibers with an orientation in the flow direction. This can be attributed to the short injection time and the resulting high shear rate, which results in a wider edge area.

Contrary to the expectations, the increase in melt temperature and the associated decrease in viscosity in samples 5 and 6 do not lead to the increase in fiber orientation in the flow direction described in Section 3.1.

Based on the results of the process-induced fiber orientation of the open hot runner system, which was characterized by means of µCT, again no clear correlation to the mechanical properties of the composites shown in Figure 4 can be concluded. This will be discussed in detail in the following section.

### 3.5. Discussion

As described in the previous paragraphs, the mechanical properties of the characterized composites could not be attributed only to one structural feature, such as the thermal load, fiber length distribution, or fiber orientation. This will be discussed in the following.

Figure 5 shows that the RCF-reinforced composites darken with increasing thermal load due to higher process temperatures and dwell times. From this, it can be concluded that with increasing thermal load from Setting 01 to Setting 06, the fiber tensile strength and consequently the tensile strength of the composites is also reduced.

Regarding the fiber lengths, it was shown that these are influenced by the material shear stress resulting from the melt temperature and shear rate. Thus, also here the fiber lengths are highest at high melt temperatures (Setting 04, 05, and 06) and thus low viscosity for both hot runner systems. For this reason, especially with the open hot runner, the average fiber lengths of Settings 05 and 06 are higher than the values of Settings 01 and 02. 

Thus, the fiber length distribution, which achieves larger values at higher temperatures, represents an opposing effect with the fiber strength, which is reduced more at high temperatures. This fact provides the explanation for the fact that the RCF-reinforced specimen of Setting 01 processed with the open hot runner system achieves the highest mechanical properties due to the lowest thermal stress on the fibers despite the lowest fiber lengths present.

As already described in Section 3.1, tensile strengths are generally higher at higher melt temperatures and high injection volume flow rates due to better fiber orientation. This effect with e.g., higher injection velocities at Settings 02-06 and higher melt temperatures at Settings 04, 05, and 06 thus also partially counteracts the effects of fiber length distribution and fiber strength.

**Figure 11:**
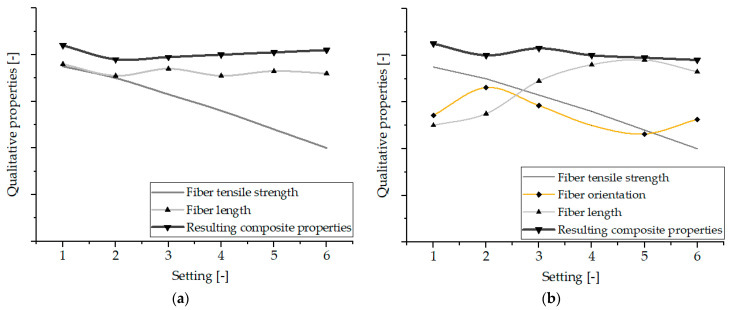
Qualitative curves of structural properties (fiber tensile strength, fiber length, and fiber orientation) and resulting composite properties of the characterized setting for the valve gate (**a**) and the open hot runner system (**b**).

The qualitative curves in Figure 11 from the values of fiber strength [74] and average fiber length for the valve gate hot runner and additionally the fiber orientation (Θ-values between 5° and 15°) for the open hot runner system of the various settings show that a curve qualitatively composed of these variables describes very well the course of the tensile strengths of the composites from Figure 4.

## 4. Conclusions

In summary, the investigations carried out here showed that cellulose fiber-reinforced composites from PP can be processed very well with various hot runner systems in the injection molding process.

Regarding the influence of hot runner processing, it was shown that the tensile strength of the GF-composites and the RCF-composites is about 20% lower than the reference samples even at the lowest thermal load (Setting 01). 

As expected, the tensile strengths of the RCF composites decrease with high thermal load (Setting 06) up to 30% for the valve gate hot runner and 22% for the open hot runner. The elongation at break of the RCF composites at Setting 06 is about 28% (valve gate) and 10% (open hot runner) lower than for the reference specimen.

These differences are partly due to the resulting fiber length distributions, whose median in both hot runner systems is 20% lower than that of the reference batches for the GF composites and only about 5% lower for the RCF composites. With regard to the fiber orientation, it was found that the different parameter settings have an influence on the orientation of the RCF in the core and edge areas. Especially at the higher shear rates, the fibers in the edge region are more oriented in the flow direction and thus also in the loading direction.

Summarized it was shown that the resulting mechanical properties of the composites are not only attributable to one structural property but are rather based on a combination of several process-related properties. The influence of the process parameters was also presented, showing the temperature-time influence on the properties of the RCF composites. From this, it can generally be deduced that the thermal load as a combination of temperature and cycle time should be kept as low as possible.

However, in the context of this temperature-time influence, it also shows that the process window for achieving good mechanical properties is quite large. This increases the fault tolerance in a later series production and thus also the acceptance of the partially bio-based composites. Thus, the results represent a further step towards the possible series production of components made from alternative material systems by means of injection molding.

## Figures and Tables

**Table 1:** Lengths and mechanical properties of different cellulosic fibers [51,52,53].

	Initial Length [mm]	Diameter [µm]	Young´s Modulus [GPa]	Tensile Strength [MPa]	Elongation [%]
**Cordenka Rayon** **(Viscose Fiber)**	2.3	12	22	778	13
**Tencel FCP (Lyocell Fiber)**	0.4–6	f	15	556	11
**Arbocel BC1000 (pure Cellulose)**	0.7	20	[-] *	[-] *	[-] *

* not measurable due to short fiber length.

**Table 2:** Process temperatures of twin screw compounding.

	Feeding Zone	Zone 1	Zone 2	Zone 3	Zone 4	Zone 5	Zone 6	Zone 7
**Temperature [°C]**	200	200	180	180	160	140	140	160

**Table 3:** Process temperatures of injection molding.

	Feeding Zone	Zone 1	Zone 2	Zone 3	Zone 4
Temperature [°C]	40	200	200	200	200

**Table 4:** Process parameters of injection molding.

Material	Setting	Hot Runner Temperature [°C]	Cooling Time [s]	Injection Time [s]	Holding Pressure [bar]	Holding Time [s]	Dwell Time [s]
PP 20GF	01	200	8	0.50	300	3	16
PP 20RCF	01	200	8	0.65	300	3	16
PP 20RCF	02	200	8	0.30	300	3	16
PP 20RCF	03	200	20	0.30	300	3	28
PP 20RCF	04	220	20	0.30	300	3	28
PP 20RCF	05	240	20	0.30	300	3	28
PP 20RCF	06	240	40	0.30	300	3	48

**Table 5:** XFiber extension settings for the individual evaluated samples.

XFiber Parameter		Setting				
		01	02	03	05	06
Cylinder length	[μm]	38	38	38	38	38
Angular sampling	[-]	5	5	5	5	5
Mask cylinder radius	[μm]	8.3	8.3	8.3	8.3	8.3
Outer cylinder radius	[μm]	6.3	6.3	6.3	6.3	6.3
Minimum seed correlation	[-]	203	203	203	196	198
Minimum continuation quality	[-]	140	123	107	98	89
Direction coefficient	[-]	0.4	0.4	0.4	0.4	0.4
Minimum distance	[μm]	3	3	3	3	3
Minimum length	[μm]	38	38	38	38	38

**Table 6:** Percentile values (10th, 50th and 90th) from the fiber length distributions of the characterized composites.

Composite	System	x10 [µm]	x50 [µm]	x90 [µm]
Reference GF	cold runner	108.6	436.1	1311.3
Reference RCF	cold runner	143.1	411.7	832.9
PP 20GF 01	open hot runner	110.4	357.1	742.0
PP 20RCF 01	open hot runner	90.9	303.4	979.9
PP 20RCF 02	open hot runner	98.9	324.6	1033.8
PP 20RCF 03	open hot runner	103.3	386.9	1155.4
PP 20RCF 04	open hot runner	94.8	390.9	1233.9
PP 20RCF 05	open hot runner	102.0	422.7	1192.9
PP 20RCF 06	open hot runner	98.7	391.9	1232.2
PP 20GF 01	valve gate hot runner	111.4	361.1	747.1
PP 20RCF 01	valve gate hot runner	96.5	404.8	1444.3
PP 20RCF 02	valve gate hot runner	106.9	384.9	1311.5
PP 20RCF 03	valve gate hot runner	106.4	399.9	1324.1
PP 20RCF 04	valve gate hot runner	102.4	371.8	1310.9
PP 20RCF 05	valve gate hot runner	90.9	398.5	1444.5
PP 20RCF 06	valve gate hot runner	97.6	388.6	1392.8

## Data Availability

Not applicable.
